# Glycosylation Status of CD43 Protein Is Associated with Resistance of Leukemia Cells to CTL-Mediated Cytolysis

**DOI:** 10.1371/journal.pone.0152326

**Published:** 2016-03-24

**Authors:** Kana Hasegawa, Satomi Tanaka, Fumihiro Fujiki, Soyoko Morimoto, Katsuhiko Nakano, Hiroko Kinoshita, Atsushi Okumura, Yuka Fujioka, Rika Urakawa, Hiroko Nakajima, Naoya Tatsumi, Jun Nakata, Satoshi Takashima, Sumiyuki Nishida, Akihiro Tsuboi, Yoshihiro Oka, Yusuke Oji, Eiji Miyoshi, Takako Hirata, Atsushi Kumanogoh, Haruo Sugiyama, Naoki Hosen

**Affiliations:** 1 Department of Cancer Immunology, Osaka University Graduate School of Medicine, Suita, Osaka, Japan; 2 Department of Functional Diagnostic Science, Osaka University Graduate School of Medicine, Suita, Osaka, Japan; 3 Department of Cancer Immunotherapy, Osaka University Graduate School of Medicine, Suita, Osaka, Japan; 4 Department of Respiratory Medicine, Allergy and Rheumatic Disease, Osaka University Graduate School of Medicine, Suita, Osaka, Japan; 5 Department of Immunopathology, WP1 Immunology Frontier Research Center, Osaka University, Suita, Osaka, Japan; 6 Department of Cancer Stem Cell Biology, Osaka University Graduate School of Medicine, Suita, Osaka, Japan; 7 Department of Fundamental Biosciences, Shiga University of Medical Science, Otsu, Shiga, Japan; Medical College of Wisconsin, UNITED STATES

## Abstract

To improve cancer immunotherapy, it is important to understand how tumor cells counteract immune-surveillance. In this study, we sought to identify cell-surface molecules associated with resistance of leukemia cells to cytotoxic T cell (CTL)-mediated cytolysis. To this end, we first established thousands of monoclonal antibodies (mAbs) that react with MLL/AF9 mouse leukemia cells. Only two of these mAbs, designated R54 and B2, bound preferentially to leukemia cells resistant to cytolysis by a tumor cell antigen–specific CTLs. The antigens recognized by these mAbs were identified by expression cloning as the same protein, CD43, although their binding patterns to subsets of hematopoietic cells differed significantly from each other and from a pre-existing pan-CD43 mAb, S11. The epitopes of R54 and B2, but not S11, were sialidase-sensitive and expressed at various levels on leukemia cells, suggesting that binding of R54 or B2 is associated with the glycosylation status of CD43. R54^high^ leukemia cells, which are likely to express sialic acid-rich CD43, were highly resistant to CTL-mediated cytolysis. In addition, loss of CD43 in leukemia cells or neuraminidase treatment of leukemia cells sensitized leukemia cells to CTL-mediated cell lysis. These results suggest that sialic acid-rich CD43, which harbors multiple sialic acid residues that impart a net negative surface charge, protects leukemia cells from CTL-mediated cell lysis. Furthermore, R54^high^ or B2^high^ leukemia cells preferentially survived *in vivo* in the presence of adaptive immunity. Taken together, these results suggest that the glycosylation status of CD43 on leukemia is associated with sensitivity to CTL-mediated cytolysis *in vitro* and *in vivo*. Thus, regulation of CD43 glycosylation is a potential strategy for enhancing CTL-mediated immunotherapy.

## Introduction

The recent success of cancer immunotherapy in solid tumors, e.g., checkpoint antibodies such as anti-CTLA-4 [1] or anti-PD-1 [2], highlights the importance of enhancing cytotoxic T cell (CTL)-mediated immune surveillance in elimination of tumor cells. Efficient immunotherapy for leukemia also needs to be developed. Several types of immunotherapies that induce a CTL response to leukemia-associated antigens (e.g. cancer vaccines) have been developed. Some of these immunotherapies have shown promising results [3–5], but still need improvement.

To improve CTL-mediated immunotherapy for leukemia, in addition to enhancing the CTL response to tumor antigen, it is also important to sensitize leukemia cells to CTL-mediated cytolysis. In solid tumors, tumor cells counteract CTLs by up-regulating certain surface molecules such as B7-H1 [6] and HLA-G [7] and shed surface molecules such as MIC, the ligand for NKG2D in soluble form [8]. Inhibition of these molecules is a strategy for sensitization of tumor cells to CTL-mediated cytolysis. However, the identities of the cell-surface molecules that protect leukemia cells from CTL-mediated cell lysis remain unknown.

To identify cell-surface molecules expressed on myeloid leukemia cells and associated with resistance to CTL-mediated cytolysis, we used MLL/AF9-induced mouse leukemia cells. MLL/AF9, a fusion gene generated by the t(9;11) translocation [9] that is responsible for a subset of human acute monocytic leukemia, can transform hematopoietic progenitor cells (HPCs) [10], and the resultant MLL/AF9 leukemia cells can be expanded without limit *in vitro* in the presence of cytokines. First, we established a number of mAbs that reacted with MLL/AF9 leukemia cells. We then screened for mAbs that were specific for cytolysis-resistant leukemia cells, which were obtained by co-culturing immunogenic antigen-expressing MLL/AF9 leukemia cells with antigen-specific CTLs. Ultimately, we isolated two mAbs specific for cytolysis-resistant leukemia cells, and then identified the antigens they recognized.

## Materials and Methods

### Animals

C57BL/6 mice (from 6- to 8- week old, female) were purchased from CREA Japan (Tokyo, Japan). CD43^-/-^ mice were kindly provided from Takako Hirata (Shiga University of Medical Science). OT-1 transgenic mice were obtained from the center of animal resources in Kumamoto University. Lewis rats (4 weeks old) were purchased from Charles River (Kanagawa, Japan). All animal experiments in this study were approved by the administrative panel on laboratory animal care in Osaka University.

### Retroviral transduction of BM progenitor cells and transplantation

MLL-AF9 cDNA [9] and OVA cDNA [11], which were kindly gifted from Cleary ML (Stanford University) and Bevan MJ (University of Washington), were subcloned into MSCV-Neo vector and MSCV-IRES-GFP vector, respectively. Retroviral stocks were produced by transient transfection of retroviral vectors to the Plat-E packaging cell line [12] (a kind gift from Kitamura T, Tokyo University) using Lipofectamine 2000 (Invitrogen, Carlsbad, CA, USA). C-kit^+^ BM cells were purified from 4- to 8-week-old mice using anti-c-kit microbeads (Miltenyi Biotec, Auburn, CA), cultured overnight in RPMI 1640 medium supplemented with 10% fetal calf serum, 10 ng/ml SCF, 10 ng/ml IL-3, and 10 ng/ml IL-6 (Pepro Tech, Rocky Hill, NJ), and then infected with MLL/AF9-Neo retroviral supernatants in the presence of 4 μg/ml Polybrene for 24 hours. Two days after the infection, cells were plated in methylcellulose medium (M3231, Stem Cell Technologies, Vancouver, BC) containing 10 ng/ml SCF, 10 ng/ml IL-6, 10 ng/ml GM-CSF, 10 ng/ml IL-3, and 400μg/ml G418 (Roche, Mannheim, Germany). After 5 days of culture, colonies were pooled, and then 10^4^ cells were replated in the same medium. At the end of the third round culture, a colony was plucked up from methylcellulose and transferred to liquid culture in the media containing 10 ng/ml SCF, 10 ng/ml IL-3, and 10 ng/ml IL-6. The resultant MLL/AF9 leukemia cells were infected with MSCV-OVA-ires-EGFP virus, and then EGFP^+^ cells were FACS-sorted using FACS Aria II (BD Biosciences, San Jose, CA). Leukemia cells expressing variable levels of OVA-IRES-GFP were FACS-sorted and used as appropriate for each experiment. For example, when enhancement of cytotoxicity by CTLs was expected, leukemia cells were used that expressed OVA-IRES-GFP at threshold levels to induce CTL activation. Establishment of mouse MLL/AF9 leukemia cells was approved by the institutional committee for recombinant DNA experiments of Osaka University. Immortalized hematopoietic progenitor cells expressing MLL/AF9 (and OVA) were expanded *in vitro* and transplanted into recipient mice by retro-orbital injection. To minimize suffering and distress, mice were subjected to inhaled anesthesia (isoflurane) prior to injection of leukemia cells. The health status of mice transplanted with leukemia cells was carefully examined twice a week. Mice were sacrificed by excess anesthesia with pentobarbital prior to analysis.

### Generation of mAbs

Four-week-old Lewis rats were immunized by footpad injection of MLL/AF9 leukemia cells twice a week. To minimize suffering and distress, rats were subjected to inhaled anesthesia (isoflurane) prior to injection of leukemia cells. The health status of rats transplanted with leukemia cells was carefully examined twice a week. After the fourth immunization, the rats were euthanized with excess pentobarbital. Lymphocytes from popliteal lymph nodes were fused with SP2/0 mouse myeloma cells by using PEG (Roche Applied Science, Basel, Switzerland). To identify MLL/AF9 leukemia cell–specific mAb-secreting hybridoma clones, MLL/AF9 leukemia cells were stained with hybridoma supernatants, followed by PE-conjugated anti-rat IgG antibody (BioLegend, San Diego, CA, USA), and then analyzed by FACS. Hybridoma clones producing mAbs that bound to MLL/AF9 leukemia cells were selected and stocked for further analysis.

### ^51^Cr releasing assay

Splenocytes from the OT1 transgenic mice were cultured in the media containing 10% FBS, 45% RPMI1640 medium, 45% AIM-V, 65IU/ml IL2, and 7.5 μg/ml SIINFEKL peptide for 5 days. Then, CD8^+^ T cells purified using CD8 T cell enrichment kit (BD Biosciences) were used as effector cells. Target cells were labeled with Chromium-51 (^51^Cr) by incubating for 90 minutes, and then co-cultured with the OVA-specific CD8^+^ T cells for 4 hours. An aliquot of supernatant was removed, and the amount of radioactivity was measured with a gamma counter. Percent of specific lysis was calculated as follows: [(experimental release–spontaneous release) / (maximum release–spontaneous release)] × 100.

### Flow cytometry

Cells were treated with Human Serum AB (Gemini Bio-Products, West Sacramento, CA, USA) to block non-specific mAb binding, and then stained with the following fluorochrome-conjugated antibodies: α-MHC I (H-2Kb) (AF6-88.5.5.3: eBioscience, San Diego, CA, USA), CD45R (B220) (RA3-6B2: eBioscience), CD11b (M1/70: BioLegend), CD4 (GK1.5: BioLegend) CD8 (53–6.7: BioLegend), CD117 (c-kit) (2B8: BioLegend), Ly-6A/E (Sca-1) (E13-161.7: BioLegend), and CD43 (S11: Biolegend). Biotin-conjugated CD3, B220, Mac-1, Ly-6G/Ly-6C (Gr-1) (RB6-8C5: BioLegend), TER-119 (TER-119, BioLegend) and PerCP/Cy5.5-streptavidin (BioLegend) were used to stain lineage-positive cells in the analysis of BM cells. Cells were analyzed using a FACS Aria II or FACS Canto II (BD Biosciences). For the analysis of cytokine production, MLL/AF9 leukemia cells were co-cultured with OT-1 CD8^+^ T cells in the medium containing 10% FBS, 45% RPMI1640 medium, 45% AIM-V medium, and 5μg/ml of Brefeldin A (Sigma Aldrich, St. Louis, MO, USA) for 4 hours at 37°C. Cells were then fixed and stained for intracellular cytokines using the Cytofix/Cytoperm kit (BD Biosciences) and anti-IFN-γ mAb (XMG1.2: BD Biosciences). To determine whether the antigen epitopes were associated with glycosylation, cells were treated with 1mM benzyl-GalNac (Sigma Aldrich) for 24 hours or 250 U/ml sialidase (New England BioLabs, Ipswich, MA, USA) for one hour at 37°C, and then subjected to FACS analysis.

### Expression cloning

Expression cloning was performed as previously reported [13] with slight modifications. cDNA library was generated from MLL/AF9 leukemia cells using Superscript Choice System (Invitrogen) and BstXI adaptor (Invitrogen). cDNA fragments ranging from 1.0 to 5.0 kb were selected by Chroma spin column (Clontech, Mountain View, CA, USA) and electrophoresis on an agarose gel and subcloned into pMX retrovirus vector (a kind gift from Toshio Kitamura, Tokyo University, Tokyo, Japan). Retrovirus carrying cDNA library derived from MLL/AF9 leukemia cells was produced by transient transduction to Plat-E cells, and then infected to YB2/0 cells [13]. YB2/0 cells reacted with R54 or B2 mAbs were FACS-sorted and expanded. After third round of FACS-sorting, insert DNA was amplified from cDNA derived from the enriched cells, and sequenced.

### Statistical analysis

Statistical analysis in each experiment was performed by two-sample t-test. Differences with P < 0.05 were considered significant.

## Results

### Identification of mAbs that react preferentially with MLL/AF9 leukemia cells resistant to CTL-mediated cell lysis

**Fig 1:**
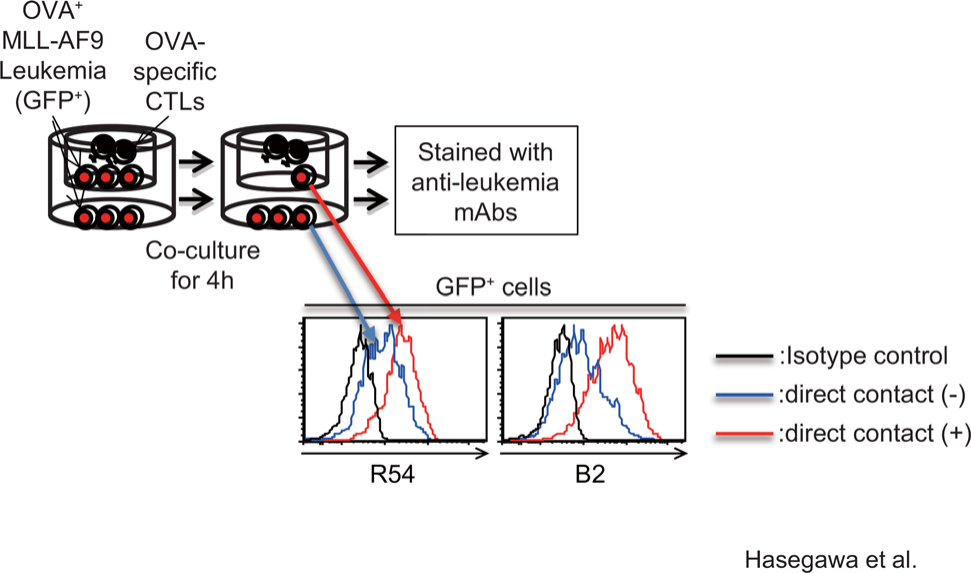
mAbs R54 and B2 preferentially react with MLL/AF9 leukemia cells that are resistant to CTL-mediated cell lysis. Scheme showing the strategies for identifying mAbs that preferentially react with MLL/AF9 leukemia cells resistant to CTL-mediated cell lysis. Histogram shows the amount of mAbs R54 or B2 bound to GFP^+^ MLL/AF9 leukemia cells after co-culture with OVA-specific T cells, with or without direct contact. The results of the isotype control did not differ between the two groups; the results obtained with leukemia cells in direct contact are shown.

MLL/AF9 leukemia cells were established by transduction of MLL/AF9 cDNA into mouse hematopoietic progenitor cells, as previously reported [14]. The resultant MLL/AF9 leukemia cells could be expanded *in vitro* without limit in the presence of SCF, IL3, and IL6. To make a library of monoclonal antibodies (mAbs) against cell-surface antigens expressed on MLL/AF9 leukemia cells, we established more than a thousand mAbs by immunizing Lewis rats with MLL/AF9 leukemia cells. To isolate leukemia cells resistant to cytolysis by CTLs, MLL/AF9 leukemia cells were transduced with ovalbumin (OVA) as a model tumor cell antigen, and then co-cultured with OVA-specific T cells derived from OT-1 transgenic mice. At the same time, leukemia cells and T cells were co-cultured without direct contact using transwell chambers. After 4 hours of co-culture, leukemia cells were stained with each clone from the anti-leukemia mAb library, and then analyzed by FACS to identify mAbs that preferentially bound to leukemia cells that had survived CTL-mediated cell lysis relative to cells that were cultured in the presence of the CTLs but without direct contact. Ultimately, we found that two mAbs, designated R54 and B2, preferentially bound to the cytolysis-resistant leukemia cells (Fig 1).

### mAbs R54 and B2 both recognize CD43 protein

**Fig 2:**
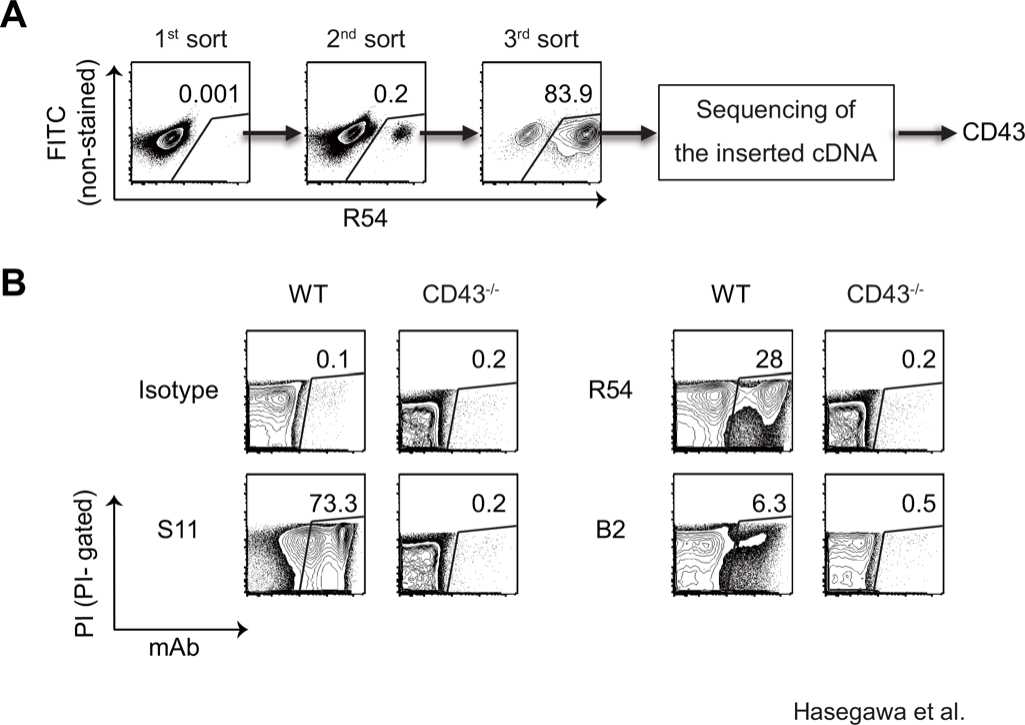
mAbs R54 and B2 both specifically recognize CD43. (A) FACS plots showing the course of the enrichment of R54-positive cells from YB2/0 cells transduced with a MLL/AF9 leukemia cell–derived cDNA library. (B) FACS analysis of the binding of S11, R54, or B2 mAb to splenocytes from wild-type or CD43-deficient mice.

Complementary DNA (cDNA) library was generated from MLL/AF9 leukemia cells, and inserted into the pMx retroviral vector. Retrovirus carrying the cDNA library was produced by transient transfection of the retroviral vector into Plat-E producer cells [12]. Next, YB2/0 cells, which were completely negative for staining with mAb R54, were infected with the retrovirus. Three days after infection, a tiny but distinct R54-positive cell population could be identified, and these cells were FACS-sorted and expanded (Fig 2A). After the third sort, almost all YB2/0 cells were R54-positive. Finally, the cDNA inserted in the retroviral vector carried by the R54-positive cells was amplified by PCR and subjected to sequencing. The inserts for R54 encoded CD43, and a similar analysis revealed that the antigen recognized by B2 was also CD43.

To confirm that R54 and B2 were specific for CD43, splenocytes from CD43-deficient mice [15] were stained with both mAbs. Although a subset of splenocytes from wild-type mice stained positively with R54 or B2, those from CD43-deficient mice were negative for R54 and B2 staining, indicating that these two mAbs specifically react with CD43 protein (Fig 2B). In addition, the percentages of B2-positive cells were much lower than the percentages of R54-positive cells. Furthermore, both R54 and B2 reacted only with subsets of splenocytes, whereas almost all splenocytes stained positively with a pre-existing anti-pan CD43 mAb (clone S11).

### The specificity of mAbs R54 and B2 differs from that of the pre-existing pan-CD43 mAb S11

**Fig 3:**
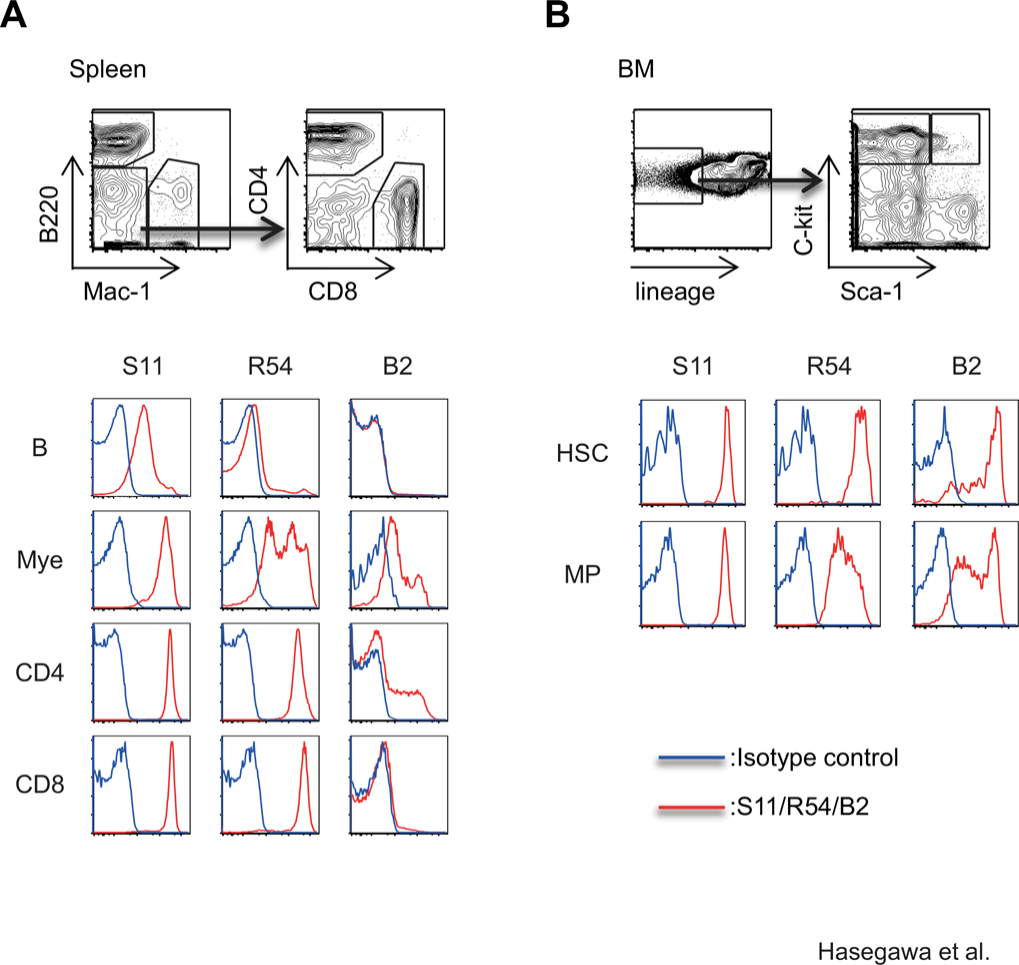
The specificities of mAbs R54 and B2 differ from that of the pan-CD43 mAb S11. (A) FACS analysis for binding of mAb S11, R54, or B2 to B, myeloid, CD4 T, and CD8 T cells in splenocytes from wild-type mice. Gating strategies for each populations are also shown. (B) FACS analysis for binding of mAb S11, R54, or B2 to hematopoietic stem cell (HSC) or myeloid progenitor cell (MP) populations of BM cells from wild-type mice.

We next compared the binding of R54 and B2 to various subpopulations of splenocytes from wild-type mice to the binding of S11. In contrast to S11, which bound to all leukocytes, R54 did not react with B cells in the spleen. In addition, whereas S11 uniformly bound to myeloid cells, myeloid cells were distinctly separated into R54^low^ and R54^high^ populations. B2 bound to myeloid cells and a subset of CD4^+^ T cells, but not to B cells or CD8^+^ T cells (Fig 3A). We also analyzed the binding of these anti-CD43 mAbs in hematopoietic stem cells (HSCs: lineage (lin)^-^, c-kit^+^, sca-1^+^) and myeloid progenitor cells (MPs: lineage (lin) ^-^, c-kit^+^, sca-1^-^) in bone marrow (BM). Although S11 uniformly bound to all HSCs and MPs, expression of R54 antigens was highest in HSCs and decreased upon differentiation into MPs. B2 antigens were expressed on HSCs and MPs at various levels, and we observed no difference in B2 antigen expression between HSCs and MPs (Fig 3B). These results clearly demonstrate that R54, B2, and S11 recognize different antigen epitopes.

### mAbs R54 and B2 are sensitive to O-glycosylation inhibitor or sialidase

**Fig 4:**
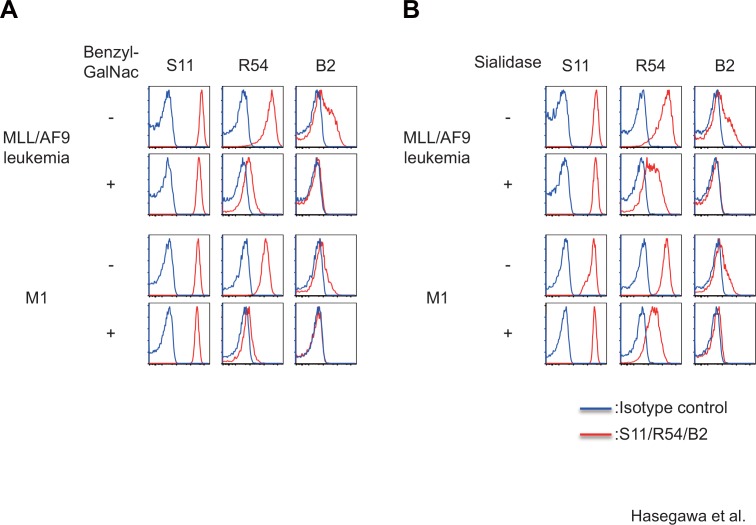
Epitopes for R54 and B2 mAbs, but S11 mAb, are sensitive to O-glycosylation inhibitor or sialidase. FACS analysis of binding of each CD43-specific mAb to MLL/AF9 leukemia cells or M1 leukemia cells treated with 1 mM benzyl-GalNac for 24 hours (A) or 250 U/ml sialidase for 1 hour (B).

Because CD43 is a heavily O-glycosylated protein, we hypothesized that R54 or B2 might recognize glycosylation-associated epitopes. To determine whether the O-glycochain is necessary for binding of each mAb, we stained MLL/AF9 leukemia cells or M1 leukemia cells treated with benzyl-GalNac, an O-glycosylation inhibitor, with each mAb and then analyzed the stained cells by FACS. Binding of R54 and B2, but not the S11, to MLL/AF9 leukemia and M1 leukemia cells was significantly reduced by treatment with benzyl-GalNac (Fig 4A). We also investigated whether the epitopes for these mAbs were sialidase-sensitive. Binding of R54 and B2, but not the S11, to MLL/AF9 leukemia and M1 leukemia cells was also reduced by the treatment with sialidase (Fig 4B). Together, these data indicate that R54 and B2, but not S11, are sensitive to inhibition of O-glycosylation or removal of sialic acid, indicating that binding of R54 and B2 reflects the glycosylation status of CD43 as well as its expression level.

### Glycosylation status of CD43 on leukemia cells is associated with sensitivity to CTL-mediated cytolysis

There was a variety in the expression levels of R54 epitope in the MLL/AF9 leukemia cells, while the leukemia cells were uniformly stained with pan-CD43 mAb S11, and could not be separated into two populations according to intensity of S11 staining (Fig 4). These results suggest that CD43 protein are uniformly expressed on leukemia cells, but the amount of sialic acid residues on CD43 protein vary among the leukemia cells.

**Fig 5:**
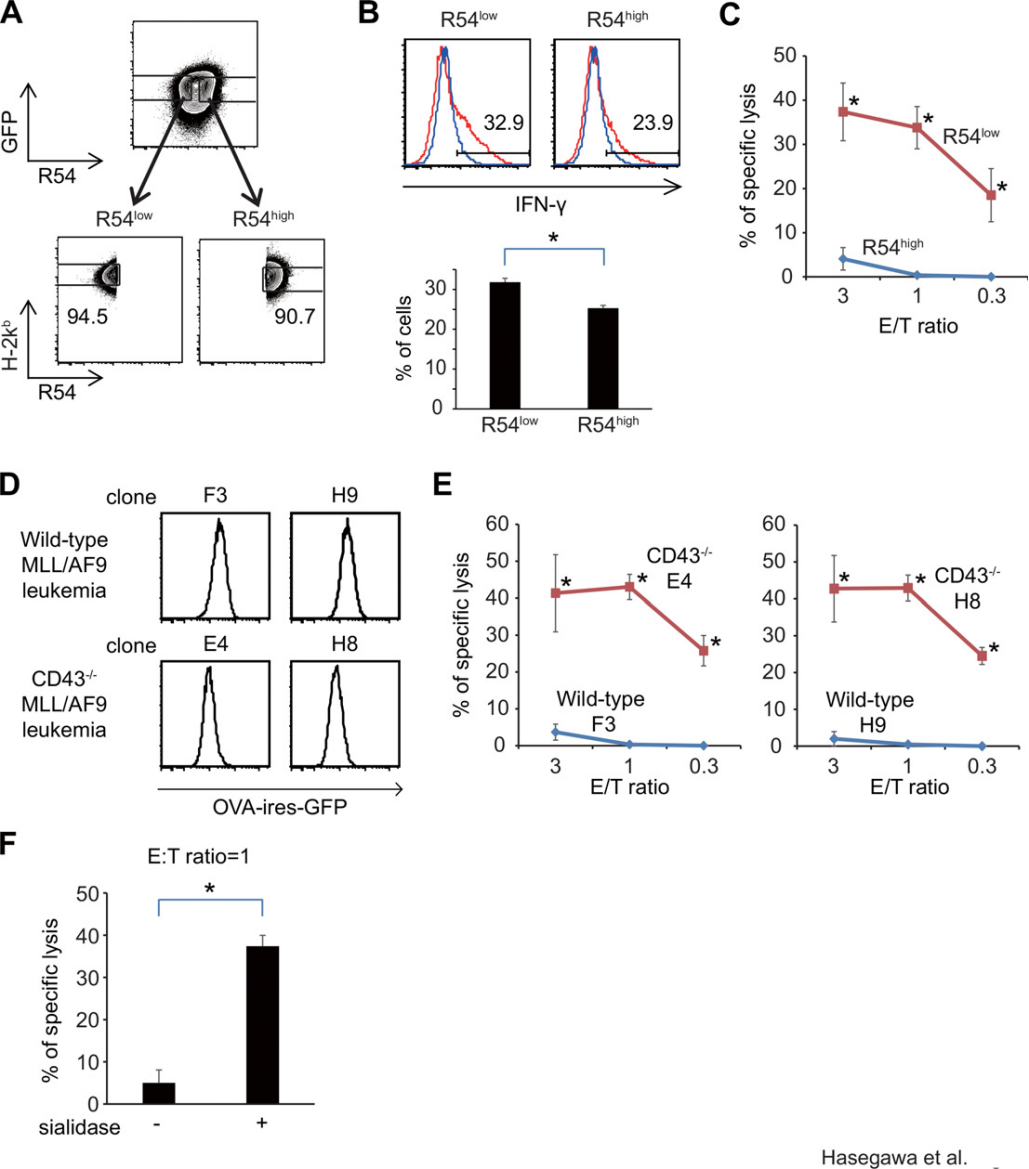
Glycosylation status of CD43 on leukemia cells is associated with sensitivity to CTL-mediated cytolysis. (A) Gating strategies for FACS-sorting the R54^high^ and R54^low^ subpopulations of OVA-expressing MLL/AF9 leukemia cells. (B) FACS analysis of intracellular IFN-γ in OT-1 T cells after co-culture with either R54 ^high^ or R54^low^ MLL/AF9 leukemia cells. IFN-γ expression in CD8^+^ T cells is shown. (C) ^51^Cr cytotoxicity assay with OT-1 T cells, using either R54 ^high^ or R54^low^ leukemia cells as targets. (D) FACS analysis of OVA-IRES-GFP expression levels in MLL/AF9-OVA leukemia clones derived from c-kit^+^ BM cells of the wild type or CD43^-/-^ mouse (E) ^51^Cr cytotoxicity assay with OT-1 T cells, using either the wild type or CD43^-/-^ leukemia cells as targets (F)^51^Cr cytotoxicity assay with OT-1 T cells, using leukemia cells with or without sialidase treatment (E/T ratio = 1).

To confirm that leukemia cells expressing the R54 epitope at high levels are resistant to OVA-specific CTLs, we FACS-sorted OVA-expressing MLL/AF9 leukemia cells into R54^high^ and R54^low^ subpopulations expressing comparable levels of OVA-ires-GFP and H-2k^b^, and then co-cultured them with OVA-specific T cells (Fig 5A). After 4 hours of co-cultivation, the frequencies of IFN-γ^+^ cells among CD8^+^ T cells were significantly higher in samples co-cultured with R54^low^ leukemia cells than in those co-cultured with R54^high^ cells (31.8 ± 1.0% vs. 25.3 ± 0.7%, p<0.05) (Fig 5B). The difference between R54^high^ and R54^low^ leukemia cells was more striking in a ^51^Cr cytotoxicity assay: R54^low^ leukemia cells were significantly more sensitive than R54^high^ cells to lysis by CTLs. Even at low effector/target (E/T) ratio (0.3), 18.5 ± 6.0% of R54^low^ were lysed by OVA-specific CTLs, while R54^high^ leukemia cells were not killed (p<0.05, Fig 5C). Binding of R54 to leukemia cells was not likely to cause the resistance to CTL-mediated cytolysis, because leukemia cell lysis by CTLs were not suppressed by addition of R54 to the cytotoxicity assay. The percentages of leukemia cells lysed by OVA-specific CTLs in the presence of isotype control Ab were similar to those in the presence of R54 mAb (50.6 ± 1.4% vs. 60.7 ± 1.2% (E/T ratio = 10)) (S1 Fig). These results suggest that the glycosylation status of CD43 on leukemia cells was correlated with sensitivity to CTL-mediated cytolysis.

To examine the functional importance of CD43 in protecting leukemia cells from cytolysis by CTLs, we established MLL/AF9-OVA leukemia cells from BM cells of wild-type or CD43-deficient mice, and subjected them to a cytolysis assay with OVA-specific CTLs. When the leukemia cells expressing low levels of OVA-ires-GFP were FACS-sorted and used for targets, almost no wild-type mice-derived leukemia cells were killed by OVA-specific CTLs (Fig 5E). By contrast, although expression levels of OVA-IRES-GFP in the CD43-deficient MLL/AF9-OVA leukemia cells were comparable to, or even somewhat lower than, those in wild-type mice-derived leukemia cells (Fig 5D), CD43-deficient leukemia cells were killed by OVA-specific CTLs. Even at low E/T ratio (0.3), substantial percentages of CD43-deficient leukemia cells (25.8 ± 4.1% (clone E4) and 24.5 ± 2.3% (clone H8)), but not the wild-type leukemia cells (0.0 ± 0.0% (clones F3 and H9)), were lysed by OVA-specific CTLs (p<0.05) (Fig 5E), indicating that CD43 protects leukemia cells from attack by CTLs. Previous work showed that CD43 has abundant sialic acid residues that impart a net negative surface charge thought to retard cell-cell interactions [16]. Consistent with this, we showed that removal of sialic acid residues by neuraminidase treatment enhanced leukemia cell lysis by CTLs (5.0 ± 3.1% vs. 37.4 ± 2.6%, p<0.05) (Fig 5F). These results suggest that sialic acid-rich CD43, which can be detected using R54 or B2, protects leukemia cells from CTL-mediated cell lysis.

### Glycosylation status of CD43 on leukemia cells is associated with selection of leukemia cells *in vivo* in the presence of adaptive immunity

**Fig 6:**
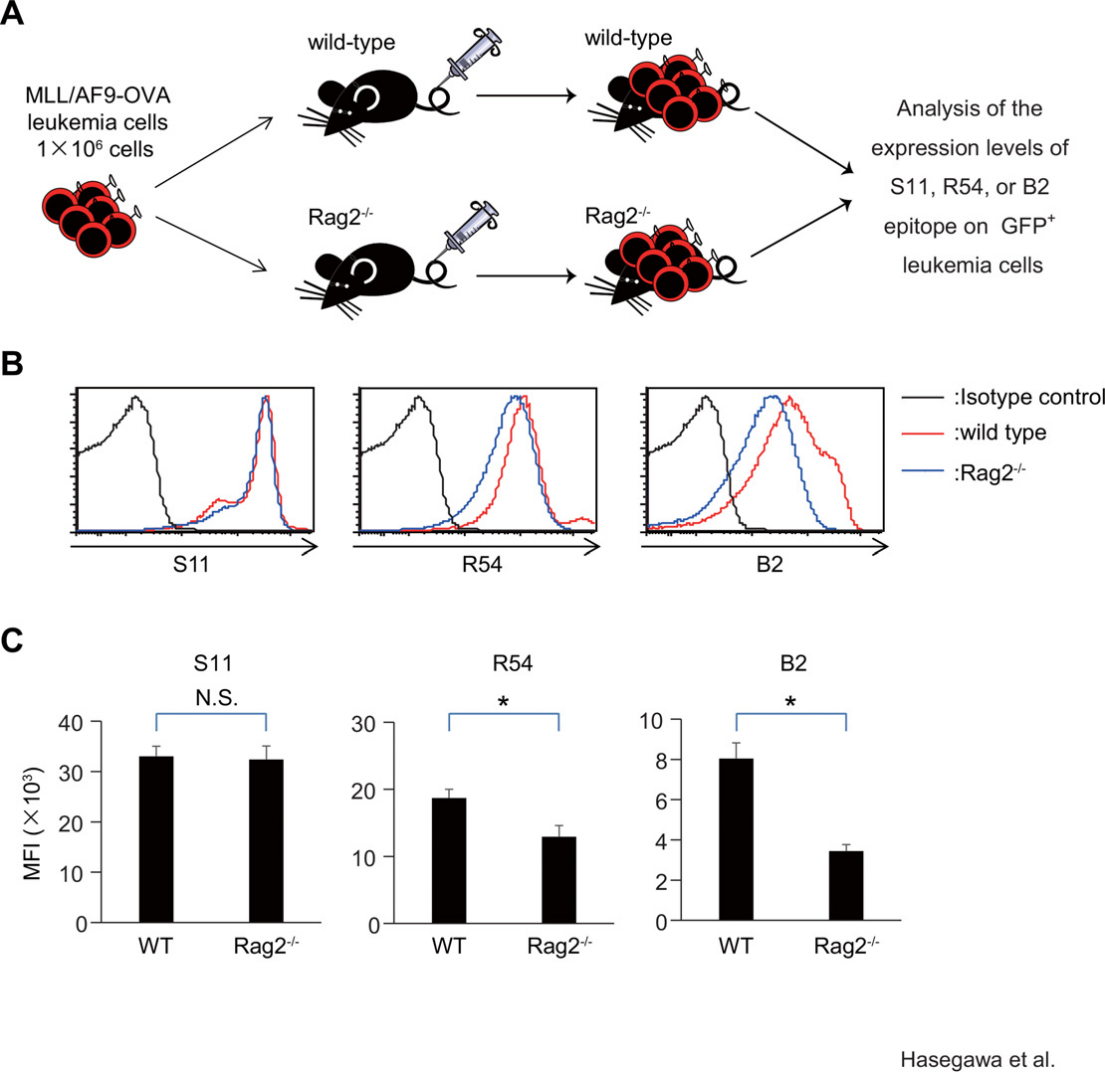
Glycosylation status of CD43 on leukemia cells is associated with selection of leukemia cells *in vivo* in the presence of adaptive immune cells. (A) Scheme showing the experimental design. (B) FACS analysis for binding of mAb S11, R54, or B2 to leukemia cells developed in wild-type or *Rag2*^-/-^ recipients (n = 5 for each genotype). Representative FACS plots are shown. (C) Bar graph showing the averages of mean fluorescence intensities (MFI). *:p<0.05, N.S.: not statistically significant.

To examine whether R54^high^ or B2^high^ MLL/AF9-OVA leukemia cells, which were less sensitive to cytolysis by CTLs *in vitro*, preferentially survived *in vivo* in the presence of an adaptive immune response, we used immune-competent leukemia models that we developed recently [17] with some modifications. One million MLL/AF9-OVA leukemia cells were transplanted into the wild-type recipients or *Rag2*^-/-^ recipients, which lack adaptive immune cells (n = 5 for each genotype). Three weeks after transplant, mice were sacrificed, and BM cells were analyzed (Fig 6A). Expression levels of S11 epitope on GFP^+^ leukemia cells were similar between the wild-type and *Rag2*^-/-^ recipients (Mean fluorescent intensity (MFI): 3.30 ± 0.19×10^4^ vs. 3.24 ± 0.27×10^4^). By contrast, expression levels of R54 and B2 epitope were significantly higher on leukemia cells from wild-type recipients than those from *Rag2*^-/-^ recipients (R54 MFI: 1.87 ± 0.13×10^4^ vs. 1.29×10^4^ ± 1.69×10^3^, p<0.05; B2 MFI: 8.04 ± 0.78 ×10^3^ vs. 3.45 ± 0.32×10^3^, p<0.05) (Fig 6B and 6C). R54^high^ or B2^high^ MLL/AF9-OVA leukemia cells preferentially survived *in vivo* in the presence of adaptive immune cells, suggesting that glycosylation status of CD43 on leukemia cells is associated with selection of leukemia cells by immune surveillance *in vivo*.

## Discussion

In this study, we established R54 and B2, new anti-CD43 mAbs that preferentially bound to MLL/AF9 leukemia cells resistant to CTL-mediated cell lysis. The epitopes for both R54 and B2 were sensitive to inhibition of O-glycosylation and removal of sialic acid. The result of staining of MLL/AF9 leukemia cells with a pan-CD43 mAb S11, which was not affected by changes of glycosylation status of CD43, suggested that CD43 protein was uniformly expressed on all MLL/AF9 leukemia cells. Taken together, these observations indicate that R54^high^ (or B2^high^) and R54^low^ (or B2^low^) leukemia cells express CD43 protein at similar levels, whereas the glycosylation status of CD43 protein differs between these cell types. Thus, glycosylation status of CD43 protein is associated with the susceptibility of leukemia cells to cytolysis by CTLs.

Previous reports showed that CD43 protein protects cells from attack by immune cells [18, 19]. In addition, the function of CD43 protein as a barrier against access by CTLs is mediated by sialic acid residues, which have negative charges and cause repulsion between cells [19, 20]. Our results are consistent with these results and also suggest that a change in glycosylation status on CD43 can be monitored by staining with sialidase-sensitive mAbs. Future studies should examine the expression of sialidase-sensitive epitopes of CD43 on human AML patients. It will be especially interesting to examine samples from AML patients who relapsed after allogeneic stem cell transplant, because the allogenic immune reaction should select CTL-resistant leukemia cells.

Our results suggested that the anti-immune effect of CD43 may be regulated through a dynamic change in sialylation status without altering CD43 protein expression levels on leukemia cells. Modulation of sialic acid expression on CD43 protein, or inhibition of the function of sialic acid residues on CD43, is therefore a potential strategy for enhancing leukemia cell lysis by CTLs. However, because sialic acid is expressed on multiple proteins on many types of cells, and has multiple functions, global regulation of sialylation process would cause multiple effects in many tissues. Therefore, it would be ideal to regulate the expression or function of sialic acid only on CD43 protein. In addition, it would be preferable to specifically target CD43 expressed on leukemia cells without affecting CD43 on other types of cells. To this end, anti-CD43 mAbs with specificity for a limited range of cell types are potentially useful. The two anti-CD43 mAbs established in this study exhibited unique specificity for subsets of hematopoietic cells. These results confirmed a previous finding that various cell type–specific antigens appear on pan-leukocyte protein CD43 as a result of post-translational modification [21–24]. In addition, tumor-specific epitopes have been identified on CD43 [23, 24]. We are now searching for a human leukemia-specific anti-CD43 mAb that has the potential to enhance leukemia cell lysis by immune cells.

In summary, we showed that glycosylation status of CD43 protein on leukemia cells is associated with sensitivity to CTL-mediated cytolysis, suggesting that regulation of glycosylation would be a promising strategy for enhancing CTL-mediated cytolysis. Various cell-type specific epitopes formed as a result of changes in CD43 glycosylation are potential targets for mAb therapy aimed at enhancing leukemia cell lysis by CTLs.

## Supporting Information

S1 Fig R54 did not suppress leukemia cell lysis by CTL-mediated cytotoxicity ^51^Cr cytotoxicity assay by OT1-CTLs was performed using MLL/AF9-OVA leukemia cells as targets in the presence of 10μg/ml of R54 or control rat IgG. N.S.: not significant.
(EPS)
